# The Neuromuscular Junction in Health and Disease: Molecular Mechanisms Governing Synaptic Formation and Homeostasis

**DOI:** 10.3389/fnmol.2020.610964

**Published:** 2020-12-03

**Authors:** Pedro M. Rodríguez Cruz, Judith Cossins, David Beeson, Angela Vincent

**Affiliations:** ^1^Nuffield Department of Clinical Neurosciences, University of Oxford, Oxford, United Kingdom; ^2^Neurosciences Group, Weatherall Institute of Molecular Medicine, University of Oxford, The John Radcliffe Hospital, Oxford, United Kingdom

**Keywords:** neuromuscular junction, myasthenia gravis, congenital myasthenic syndromes, spinal muscular atrophy, sarcopenia, MuSK, DOK7, Agrin

## Abstract

The neuromuscular junction (NMJ) is a highly specialized synapse between a motor neuron nerve terminal and its muscle fiber that are responsible for converting electrical impulses generated by the motor neuron into electrical activity in the muscle fibers. On arrival of the motor nerve action potential, calcium enters the presynaptic terminal, which leads to the release of the neurotransmitter acetylcholine (ACh). ACh crosses the synaptic gap and binds to ACh receptors (AChRs) tightly clustered on the surface of the muscle fiber; this leads to the endplate potential which initiates the muscle action potential that results in muscle contraction. This is a simplified version of the events in neuromuscular transmission that take place within milliseconds, and are dependent on a tiny but highly structured NMJ. Much of this review is devoted to describing in more detail the development, maturation, maintenance and regeneration of the NMJ, but first we describe briefly the most important molecules involved and the conditions that affect their numbers and function. Most important clinically worldwide, are myasthenia gravis (MG), the Lambert-Eaton myasthenic syndrome (LEMS) and congenital myasthenic syndromes (CMS), each of which causes specific molecular defects. In addition, we mention the neurotoxins from bacteria, snakes and many other species that interfere with neuromuscular transmission and cause potentially fatal diseases, but have also provided useful probes for investigating neuromuscular transmission. There are also changes in NMJ structure and function in motor neuron disease, spinal muscle atrophy and sarcopenia that are likely to be secondary but might provide treatment targets. The NMJ is one of the best studied and most disease-prone synapses in the nervous system and it is amenable to *in vivo* and *ex vivo* investigation and to systemic therapies that can help restore normal function.

## Introduction

The neuromuscular junction (NMJ) is a simple synapse between the motor nerve terminal and the surface of a muscle fiber sarcolemma, but is nevertheless complex in its structure and function. Most of what we know about development of the NMJ comes from work in rodents, particularly mice and evidence from work on human muscle is sparse. During development, nascent skeletal muscle fibers express AChRs on their surface and the axons of motor neurons are guided to innervate the fibers, leading to the clustering of AChRs at high density underneath the motor nerve terminals. Despite being functionally active in the embryonic stage, NMJs undergo complex postnatal maturation during the first weeks of life that consist of an increase in size, morphological changes and the development of invaginations in the subsynaptic sarcolemma. The apparent macroscopic stability of the NMJ during adulthood hides numerous mechanisms that allow the homeostasis of the neuromuscular synapse in health and disease. This review covers the molecular mechanisms underlying the development and homeostasis of the NMJ and their contribution to health and disease.

## The Organization of the NMJ

**FIGURE 1:**
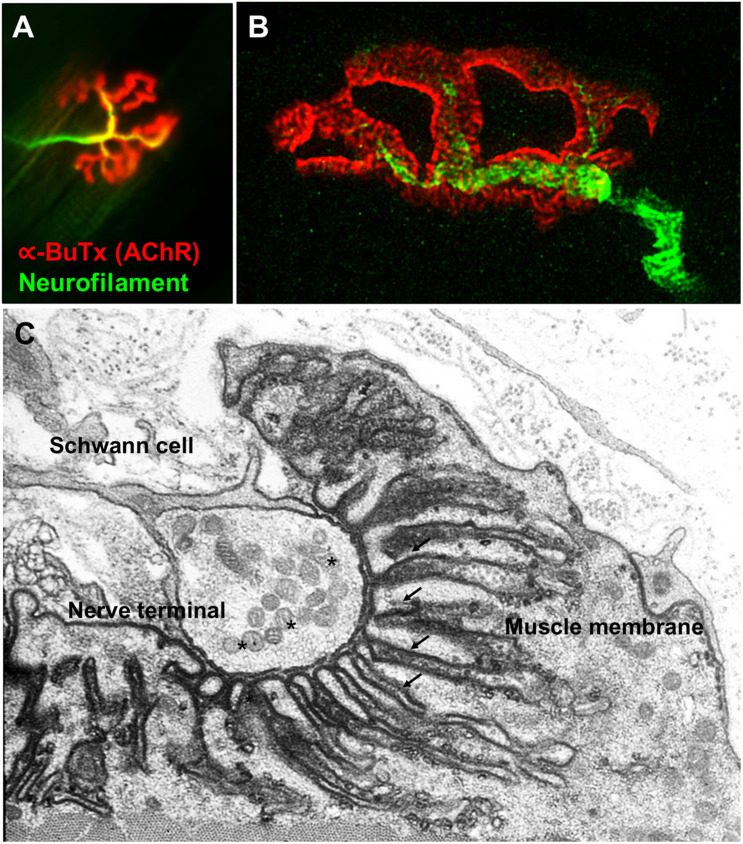
Fluorescence and electron microscopy images of the NMJ. **(A)** Fluorescence microscopy image of the NMJ showing the nerve terminal (green) innervating the muscle endplate (red) stained with fluorescently conjugated α-bungarotoxin that binds to the AChRs. **(B)** Super-resolution confocal microscopy image of the NMJ showing the postsynaptic muscle membrane and the junctional folds at the top of which AChRs are concentrated (image provided by Dr. J. Cheung). **(C)** Electron microscopy image of the NMJ. The presynaptic nerve terminal is filled with synaptic vesicles containing acetylcholine (*). The postsynaptic muscle membrane exhibits a high degree of folding which extends into the muscle sarcoplasm (arrows) in order to increase the total endplate surface. The NMJ is covered by terminal Schwann cells. Used with permission from Slater (2017).

The NMJs are very small structures (∼30 μm long) compared to the length of the muscle fibers they innervate which can be anything from less than a cm (e.g., intercostal muscle) to more than 20 cm (e.g., sartorius, the long muscle of the thigh). Typically, each skeletal muscle fibers has a single NMJ where the motor axon joins the muscle fiber. The most common classification divides the NMJ into a presynaptic terminal, a postsynaptic muscle membrane and the space that lies between called the synaptic cleft. The classic morphology of the NMJ in murine animal models is described as a pretzel-shaped structure (Figure 1). The human NMJs are typically smaller, less complex, and more fragmented than those widely studied in murine animal models (Jones et al., 2017), although they exhibit a higher degree of postsynaptic membrane folding than any other species (Slater, 2017).

### Presynaptic Terminal

Motor nerves travel from the spinal cord to skeletal muscles where they divide into terminal branches and subsequently form synaptic boutons that contact the muscle surface (Desaki and Uehara, 1981). Synaptic boutons are small protuberances found at the terminal end of motor axons. They are filled with synaptic vesicles containing the neurotransmitter ACh ready for vesicle exocytosis. The nerve terminal has complex machinery in place to allow the synthesis, exocytosis and recycling of these synaptic vesicles (Lai et al., 2017). Non-myelinating Schwann cells called perisynaptic or terminal Schwann cells (tSCs) cover the NMJ, and there is increasing evidence that they contribute to synapse formation, maintenance and repair (Feng and Ko, 2008).

### Synaptic Cleft

The synaptic cleft is the gap between the presynaptic terminal and the postsynaptic muscle membrane, which is filled with a specialized form of extracellular matrix called synaptic basal lamina (Sanes, 2003). This matrix is crucial for the alignment, organization and structural integrity of the NMJ. The main components of the basal lamina include laminins and different types of collagens (Shi et al., 2012). In particular, it is of relevance that the enzyme acetylcholinesterase (AChE), which terminates synaptic transmission by breaking down acetylcholine, is attached to the basal lamina thanks to ColQ, an NMJ-specific collagen-like tail (Ohno et al., 1998).

### Postsynaptic Muscle Membrane

The postsynaptic membrane is a specialized structure with a high degree of folding, as shown by electron microscopy studies (De Harven and Coers, 1959). Motor nerve terminals are embedded in the muscle in a gutter or primary cleft. Furthermore, there are a series of invaginations of the muscle membrane that extend into the sarcoplasm called secondary junctional folds. They increase the overall surface of the postsynaptic membrane, and the AChRs clustered at high density on the crest of these folds, juxtaposed to the presynaptic active zones. At the bottoms of the folds, voltage-gated Na+ channels are concentrated to facilitate the excitability of the postsynaptic membrane.

## Neuromuscular Transmission

The enzyme choline acetyltransferase (ChAT) synthesizes acetylcholine (ACh) from acetyl coenzyme A (AcCoA) and choline (Nachmansohn and Machado, 1943). Subsequently, acetylcholine is packed into synaptic vesicles thanks to the vesicular acetylcholine transporter (VAChT) (Roghani et al., 1994). Pools of synaptic vesicles accumulate in the presynaptic terminal near release sites termed active zones (Sudhof, 2012). Upon arrival of an action potential, voltage-gated calcium channels (VGCCs) open and Ca^2+^ inflow triggers vesicle fusion to the plasma membrane and exocytosis through the Soluble N-ethylmaleimide-sensitive factor Attachment protein Receptor (SNARE) complex (Baker and Hughson, 2016). ACh released by the presynaptic terminal binds to the ACh binding site located at the α and either δ or ε-subunits interfaces of the AChR. Upon ACh binding, the AChR subunits undergo a conformational change to open the channel creating a pore (Miyazawa et al., 2003). This event allows the influx of positively charged ions to move across the channel generating a change in the membrane potential that triggers an endplate potential (EPP). In a healthy individual, the depolarization of the postsynaptic membrane generated by the EPP is greater than the threshold needed to activate the Na_*v*_1.4 voltage-gated sodium channels and generate an action potential. The action potential spreads from the motor endplate to the rest of the sarcolemma, resulting in contraction of the muscle (Engel et al., 2015).

Importantly the NMJ is an all-or-none synapse. If the endplate potential does not reach the threshold for opening of sodium channels, or if there are insufficient sodium channels in the postsynaptic folds to generate an action potential, muscle contraction does not occur. In health, the EPP is more than sufficient to reach threshold, and the sodium channels are concentrated at the depths of the postsynaptic folds where they can be efficiently opened by the voltage change. This is the *margin of safety, or safety factor of neuromuscular transmission*, which allows the NMJ to continue to function under various physiological conditions and stresses. In disease, this margin of safety can be reduced at individual endplates, or many of them, leading to reduced neuromuscular transmission and muscle weakness (Wood and Slater, 2001). These concepts are crucial in understanding the diseases that cause muscle weakness (Table 1). Much of what is summarized below is covered in detail by other authors in this Special Issue (Cao et al., 2020; Takamori, 2020).

**TABLE 1:** Disorders of neuromuscular transmission.

Venoms and Neurotoxins		
Snake bite		
Botulism		
Tetanus		
**Myasthenia gravis**		
AChR antibodies		
MuSK antibodies		
Antibodies to clustered AChRs		
LRP4 antibodies		
**Lambert-Eaton Myasthenic Syndrome**		
VGCC antibodies		

**CMS subtype**	**Gene**	**Inheritance**

***Proteins with defined NMJ function***		
*Presynaptic*		
Choline O-Acetyltransferase	*CHAT*	AR
Unconventional myosin 9	*MYO9*	AR
PREPL	*PREPL*	AR
Vesicular ACh transporter (VAChT)	*SLC18A3*	AR
High-affinity choline transporter 1 (ChT)	*SLC5A7*	AR
Synaptosome Associated Protein 25	*SNAP25B*	AD
Synaptotagmin 2	*SYT2*	AD
Munc13-1	*UNC13-1*	AR
Rabphilin 3A	*RPH3A*	AR
Synaptobrevin 1	*VAMP1*	AR
*Synaptic*		
Collagen Type XIII Alpha 1 Chain	*COL13A1*	AR
Endplate AChE deficiency	*COLQ*	AR
Laminin α5 deficiency	*LAMA5*	AR
Laminin ß2 deficiency	*LAMB2*	AR
*Postsynaptic*		
Agrin (neuronal)	*AGRN*	AR
Primary AChR deficiency	*CHRNA, CHRNB, CHRND, CHRNE*	AR
Slow channel syndrome	*CHRNA, CHRNB, CHRND, CHRNE*	AD
Fast channel syndrome	*CHRNA, CHRNB, CHRND, CHRNE*	AR
Low conductance syndrome	*CHRNE*	AR
Escobar syndrome	*CHRNG*	AR
DOK7	*DOK7*	AR
LRP4	*LRP4*	AR
MACF1	*MACF1*	AR
MuSK	*MUSK*	AR
Plectin deficiency	*PLEC1*	AR
Rapsyn	*RAPSN*	AR
Na^+^ channel myasthenia	*SCN4A*	AR
*Ubiquitously expressed proteins*		
ALG2	*ALG2*	AR
ALG14	*ALG14*	AR
DPAGT1	*DPAGT1*	AR
GFPT1	*GFPT1*	AR
GMPPB	*GMPPB*	AR
SLC25A1	*SLC25A1*	AR

*ALG2, Alpha-1,3/1,6-Mannosyltransferase; ALG14, UDP-N Acetylglucosaminyltransferase Subunit; DOK7, Docking protein 7; DPAGT1, Dolichyl-Phosphate N-Acetylglucosaminephosphotransferase 1; GFPT1, Glutamine-Fructose-6-Phosphate Transaminase 1; GMPPB, GDP-Mannose Pyrophosphorylase B; LRP4, LDL Receptor Related Protein 4; MACF1, microtubule actin cross linking factor 1; MuSK, Muscle specific kinase; PREPL, Prolyl Endopeptidase-Like gene.*

## Disorders of the NMJ

### Venoms and Neurotoxins

Much of what we first learnt regarding the molecules at NMJ that are essential for its function, and also targets in disease came from the study of specific neurotoxins, particularly those from snake venoms on transmission at the NMJ (Vincent, 2020). Envenomation by snake bite is a very important disease globally and leads to a variety of symptoms of which, since the NMJ is accessible to the systemic circulation, defects in transmission are often early with respiratory failure (Warrell, 2010, 2019).

Botulism is an important presynaptic disorder, which is caused by a toxin produced by the anaerobic bacterium, *Clostridium botulinum*. The botulinum toxin (Botox) is a proteolytic enzyme that gets transported into the motor nerve and other nerve terminals, cleaves SNARE proteins, preventing vesicle fusion and ACh release (Schiavo et al., 2000). Tetanus toxin produced by *Clostridium tetani* is also taken up by presynaptic motor nerve terminals but travels retrogradely, via the motor neuron cell body, to the inhibitor nerve terminals of the spinal cord where it prevents the release of GABA and glycine leading to painful muscle spasms. Each of these conditions can be life-threatening due to muscle paralysis or, in the case of tetanus toxin, uncontrolled muscle contractions.

### Myasthenia Gravis

Autoantibodies against the muscle acetylcholine nicotinic receptor (AChR) cause myasthenia gravis (MG), the most common disorder of neuromuscular transmission, which is characterized by fatigable muscle weakness (Lindstrom et al., 1976; Vincent and Newsom-Davis, 1985). The AChR antibodies are predominantly IgG1 and IgG3 subclasses and lead to loss of AChRs by two main mechanisms; mainly complement activation, cross-linking and internalization of AChRs (Le Panse and Berrih-Aknin, 2013). Classic treatment is with immunosuppressive drugs and cholinesterase inhibitors to prevent the breakdown of ACh by AChE; this leads to longer duration of ACh in the synapse leading to larger and slightly prolonged EPPs.

Interestingly, there are patients in rare occasions with autoantibodies against the fetal γ-subunit of the AChR, which is present prenatally but largely replaced by the ε-subunit before birth at approximately 33 weeks (AChR γ-to-ε switch) (Missias et al., 1996). As a result, these antibodies have little effect in adults but they can cause neonatal myasthenia (Vernet-Der Garabedian et al., 1994) or arthrogryposis multiplex congenita (Oskoui et al., 2008) via maternal transfer to the fetus.

Around 10–20% of MG patients are seronegative for AChR antibodies but a variable proportion (0–70%) have antibodies to the Muscle-Specific Kinase (MuSK) (Hoch et al., 2001). MuSK autoantibodies are predominantly of the IgG4 subtype and impair agrin signaling by disrupting the interaction of MuSK with the low density lipoprotein receptor-related protein-4 (LRP4) (Koneczny et al., 2013). MuSK-MG has distinct clinical features and response to treatment, including the worsening of symptoms with anticholinesterase therapy (Evoli et al., 2003). The clinical features of this form of MG and the mechanisms by which the antibodies act can be found in *Cao et al.*, this volume (Cao et al., 2020). MG patients seronegative for MuSK and AChR antibodies by radioimmunoprecipitation assay (RIA) may have antibodies to clustered AChRs by cell-based assay (CBA) (Leite et al., 2008). These antibodies have similar pathogenic mechanisms to AChR antibodies detected by RIA (Jacob et al., 2012). They can be useful in clinical practice, especially in children, for planning treatment and to distinguish from congenital myasthenic syndromes (CMS) (Rodríguez Cruz et al., 2015). LRP4 antibodies have also been reported in seronegative MG (Zhang et al., 2012), but the detection rates are highly variable between studies, and some cases are also positive for AChR and MuSK antibodies (Higuchi et al., 2011). A further uncertainty comes from studies showing LRP4 antibodies in some patients with amyotrophic lateral sclerosis (Tzartos et al., 2014). Finally, autoantibodies to other immunogenic targets such as agrin (Gasperi et al., 2014), COLQ (Zoltowska Katarzyna et al., 2014) and cortactin (Gallardo et al., 2014) have been described. However, their pathogenic contribution and overall importance in clinical diagnosis require further study.

### Lambert-Eaton Myasthenic Syndrome (LEMS)

This disease is rarer than MG and is caused by autoantibodies against P/Q type VGCCs on the presynaptic terminal at the NMJ (Eaton and Lambert, 1957; Lennon et al., 1995). Half of LEMS patients have an associated tumor, typically small-cell lung carcinoma (SCLC), which also expresses functional VGCC. The pathogenic mechanism is from cross-linking and internalization of the VGCCs by antibodies leading to reduced expression on the presynaptic nerve terminal (Nagel et al., 1988). This results in the functional loss of VGCC in active zones, reduced Ca^2+^ entry during depolarization and a subsequent decrease in quantal content and ACh release. Complement-dependent mechanisms don’t appear to be relevant, though it is not clear why not.

### CMS

Congenital myasthenic syndromes are a group of inherited disorders caused by mutations in genes encoding for proteins that are essential for the integrity of neuromuscular transmission (Rodríguez Cruz et al., 2018). Over the years, deciphering the underlying pathogenic mechanism of CMS has helped to improve our understanding of the NMJ and refine therapeutic strategies with other drugs like 3,4-Diaminopyridine, β2−adrenergic agonists, and open-channel blockers fluoxetine and quinidine (Harper et al., 2003; Lashley et al., 2010). At present, mutations in more than 30 different genes are known to cause CMS. Most common classification is based on the location of the mutated protein (presynaptic, synaptic and postsynaptic). All CMSs present with fatigable muscle weakness, but age at onset, symptoms, distribution of weakness, and response to treatment vary, depending on the molecular mechanism resulting from the genetic defect. Future therapies may include the use of novel and more specific β2−adrenergic agonists, modulation of the Agrin-LRP4-MusK-DOK7 pathway and gene replacement therapy (Arimura et al., 2014).

### Other Neuromuscular Disorders Where the NMJ Is Involved

There is increasing evidence that muscle endplates may also be affected in motor unit disorders that are not believed to primarily affect the NMJ, including spinal muscular atrophy (SMA) and amyotrophic lateral sclerosis (ALS). SMA is an autosomal recessive disease caused by insufficient levels of survival motor neuron (SMN) protein that results in progressive loss of lower motor neurons, denervation and muscle atrophy (Ahmad et al., 2016). Studies in animal models of SMA have shown that earliest structural defects appear distally at the NMJ during postnatal maturation, even in the absence neuromuscular transmission failure or motor neuron loss (Kariya et al., 2008; Lee et al., 2011). Furthermore, patients with types 2 and 3 SMA suffer from objective motor fatigue (Wolfe et al., 2016) and 3Hz repetitive nerve stimulation shows pathological decrement in half of them (Wadman et al., 2012). Open pilot studies have reported the benefit of salbutamol in SMA patients (Pane et al., 2008). It is thought that salbutamol increases *SMN* mRNA and protein levels in SMA fibroblasts (Angelozzi et al., 2008) and patients (Tiziano et al., 2019) by promoting exon 7 inclusion in *SMN2* transcripts. However, given the remarkable effect of β2−adrenergic agonists in CMS (Lashley et al., 2010; Rodríguez Cruz et al., 2015), the effect seen in SMA patients could be at least partly related to an improvement in the NMJ function and structure.

There are several studies suggesting that ALS is a distal axonopathy where pathological changes start preclinically with denervation of the muscle endplates and then proceed in a “dying back” pattern that results in motor neuron loss (Fischer et al., 2004; Gould, 2006). While evidence supporting this hypothesis comes mainly from *SOD1*-ALS mouse models, a recent investigation suggests a direct link between NMJ signaling pathways and *FUS* (Picchiarelli et al., 2019), an ALS-associated gene whose dominant mutations cause aggressive forms of the disease. Recent studies have shown that modulation of agrin signaling by AAV-DOK7 gene therapy (Miyoshi et al., 2017) and MuSK stimulation (Cantor et al., 2018) can increase motor activity and lifespan of the *SOD1-G93A* ALS mouse model by slowing muscle denervation. Other disorders where NMJ structural defects could play a role comprise autosomal dominant Emery-Dreifuss muscular dystrophy (AD-EDMD) (Méjat et al., 2009) and some forms of Charcot-Marie-Tooth disease (CMT) (Sleigh et al., 2014). Finally there are some congenital myopathies with secondary neuromuscular transmission abnormalities (Rodríguez Cruz et al., 2014) where achieving a precise diagnosis is important as patients could benefit from symptomatic treatment with anticholinesterases.

### Ageing and Sarcopenia

The progressive decline in muscle mass and function related to ageing is known as sarcopenia. The mouse NMJ undergo dramatic structural changes with ageing in the form of increased fragmentation of endplates (Valdez et al., 2010). It is thought that this could be secondary to degeneration and regeneration of muscle fibers at the neuromuscular synapse (Li et al., 2011). Interestingly, overexpression of neurotrypsin in motoneurons destabilizes NMJs by increasing the proteolytic cleavage of agrin (Bolliger et al., 2010) and installs a phenotype compatible with sarcopenia in young adult mice (Bütikofer et al., 2011). Stabilization of the NMJs could represent a potential therapy for sarcopenia as shown by the injection of a soluble fragment of neuronal agrin (NT- 1654) in neurotrypsin overexpressing mice (Hettwer et al., 2014). Another study showed that sarcoglycan alpha reduces NMJ decline in aged mice by stabilizing LRP4 (Zhao et al., 2018). Remarkably, caloric restriction and exercise was shown to mitigate age-related changes in mouse NMJs, which opens the door to non-pharmacological interventions (Valdez et al., 2010). These observations have linked sarcopenia with the deterioration of the NMJ structure. However, a recent study showed that human NMJs, in contrast to mice, are remarkably stable throughout adult life with lack of age-related remodeling signs in the muscles tested (Jones et al., 2017). Therefore, there may be significant variability in age-related events among muscles. Furthermore, a key question that remains unanswered is whether the age-related NMJ decline contributes to or results from sarcopenia.

## Molecular Mechanisms Involved in Synapse Formation and Maintenance

Multiple mechanisms govern the assembly and homeostasis of the neuromuscular synapse as described earlier. This section will cover in greater detail the best-defined pathways and the clinical impact when perturbed. First, it is worth visualizing the NMJ in relation to the muscles that it regulates to understand that this tiny synapse has to function correctly to control the timing and efficiency of that muscle (Figure 1).

### The Agrin-LRP4-MuSK Signaling Pathway

**FIGURE 2:**
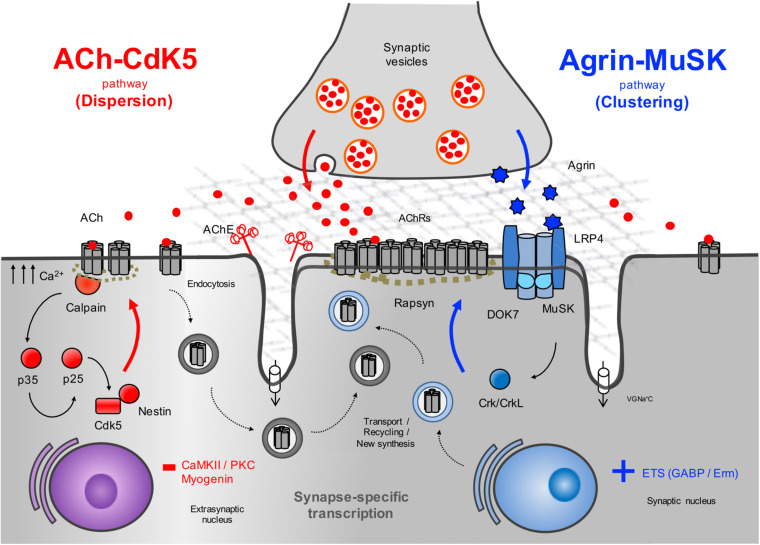
Schematic representation of the AChR clustering and dispersal pathways. Upon the release of agrin by the nerve terminal, agrin binds to LRP4 resulting in MuSK activation (Kim et al., 2008) and recruitment of DOK7 and Crk/CrkL (Okada et al., 2006) that further stimulates MuSK activation. The signal is propagated downstream, which results in the clustering of the AChRs by the cytoplasmic anchoring protein rapsyn. By contrast, ACh disperses AChR clusters not stabilized by agrin signaling. A cyclin-dependent kinase (CdK5) mechanism is thought to drive this pathway through the interaction of rapsyn and the calcium-dependent protease calpain (Chen et al., 2007). Calpain activity promotes the cleavage of p35 to p25 (Patrick et al., 1999), an activator of CdK5. On the other hand, rapsyn behaves as a calpain suppressor, thus stabilizing AChR clusters. It is thought that on synaptogenic induction, new synthesized AChRs may be transported and delivered to the nascent postsynaptic sites for insertion, together with AChRs endocytosed from the spontaneous AChR clusters. Synapse specific transcription in subsynaptic nuclei by different transcription factors and specific promoter elements in synaptic genes such as GABP (Schaeffer et al., 1998) and Erm (Hippenmeyer et al., 2007) is also key to achieve a high concentration of AChRs in synaptic sites. VGNa+C, volgated-gated Na+ channel.

**FIGURE 3:**
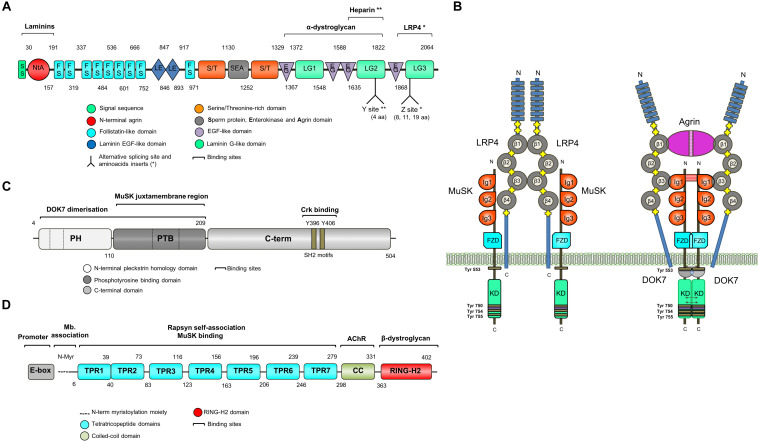
Schematic representation of key NMJ proteins. **(A)** Agrin (RefSeq NP_001292204.1) is a large proteoglycan (>200 KDa) with multiple domains that binds to laminins through the N-terminal domain, and to LRP4 and α-dystroglycan via C-terminus. Two of the three laminin G-like domains are required for binding to α-dystroglycan. Agrin mRNA undergoes cell-specific alternative splicing at different sites (Ferns et al., 1993). When agrin is produced by motor neurons, the Z site contain amino acid inserts specifically required for MuSK activation. **(B)** The binding of agrin to the N-terminal region of LRP4 induces a conformational change (active state) promoting the binding between LRP4 and the first IgG-like domain of MuSK and the formation of a tetrameric complex (Zhang et al., 2011). This results in MuSK activation via dimerization and trans-autophosphorylation of tyrosine residues within the cytoplasmic region (Schlessinger, 2000). The increase in the catalytic activity creates active binding sites for other proteins such as DOK7, leading to in full kinase activation and amplification of the signal downstream. The composition of LRP4 includes 8 Low density lipoprotein Class A (LDLa) domains (blue) at the N terminus, followed by 4 YWTD ß-propeller domains (gray) bounded by epidermal growth factor-like modules (yellow) and a short C-terminal domain (Springer, 1998). LRP4 self-associates and interacts with MuSK in the absence of agrin (inactive state), but is not capable of activating MuSK (Kim et al., 2008). **(C)** DOK7 (RefSeq NM_173660.4) is composed of an N-terminal pleckstrin homology (PH) domain, a phosphotyrosine-binding (PTB) domain and a C-terminus. Dashes are used to mark the different exons. **(D)** Rapsyn (RefSeq NP_005046.2) is composed of a N-terminal myristoylation moiety (N-Myr) necessary for submembrane localization; seven tetratricopeptide (TRP) domains responsible for rapsyn self-association and MuSK binding (Ramarao et al., 2001; Lee et al., 2008); a coiled-coil domain that binds to the cytoplasmic loops of AChRs (Lee et al., 2008), and a RING-H2 domain that interacts with the dystroglycan complex and links with the cytoskeleton (Apel et al., 1995).

The agrin signaling pathway is essential for both NMJ formation (DeChiara et al., 1996; Gautam et al., 1996; Okada et al., 2006; Weatherbee et al., 2006) and maintenance (Kong et al., 2004; Barik et al., 2014; Tezuka et al., 2014; Eguchi et al., 2016; Figure 2). Several genetic and autoimmune disorders affecting this pathway are known to cause disease (Liyanage et al., 2002). Agrin is a large proteoglycan (>200 KDa) with multiple domains that binds to laminins through the N-terminal domain, and to LRP4 and α-dystroglycan via its C-terminus (Figure 3A). A neuronal isoform of agrin, generated by alternative splicing to introduce eight additional amino acids at the Z site, is secreted from the presynaptic terminal into the basal lamina as the first step in the AChR clustering pathway (Ferns et al., 1993). Following its release, agrin binds to LRP4 on the postsynaptic muscle membrane, and this, in turn, activates MuSK (Zhang et al., 2008). LRP4 is able to self-associate and interact with MuSK independently of agrin (inactive state) but is not capable of activating MuSK (Kim et al., 2008).

MuSK is made up of three IgG-like domains, a frizzled domain (FzD), a short transmembrane (TM) and a juxtamembrane (JMR) region, a kinase domain (KD), and a short C-terminal tail (Hubbard and Gnanasambandan, 2013; Figure 3B). MuSK kinase activity is strictly regulated to limit ligand-independent activation primarily through the juxtamembrane region and the activation loop (Till et al., 2002). The interaction between the juxtamembrane region and the kinase core inhibits kinase activity. The activation loop adopts a pseudo-substrate conformation that occupies the active site cleft of the kinase domain and impedes ATP binding thus blocking kinase activation (Till et al., 2002). Also, MuSK activation is regulated by the muscle tyrosine phosphatase Shp2 (Madhavan et al., 2005). All of these regulatory mechanisms seem critical for the control of postsynaptic differentiation at the NMJ (Madhavan and Peng, 2005), but they are by no means the only molecules involved, and more could be identified in the future.

The formation of a tetrameric complex between agrin and LRP4 increases the binding of LRP4 to the first IgG-like domain of MuSK (Zong et al., 2012), leading to MuSK activation via dimerization and trans-autophosphorylation of specific tyrosine residues within the cytoplasmic region (Schlessinger, 2000). Phosphorylation of Tyr553 destabilizes the juxtamembrane conformation that prevents the phosphorylation of the activation loop and the creation of an active binding site for downstream of kinase-7 (DOK7), a cytoplasmic adaptor of MuSK with a phosphotyrosine binding domain (PTB). The subsequent transphosphorylation (Tyr750, Tyr754, and Tyr755) of the activation loop results in full kinase activation.

### Intracellular Pathways Downstream of MuSK

The agrin-LRP4-MuSK signal is propagated downstream leading to the clustering of AChRs by the scaffold protein rapsyn. However, how the signal is transduced from MuSK to rapsyn is not understood, with the exception of the key adapter protein DOK7, which is required for full MuSK activation (Inoue et al., 2009). DOK7 is composed of an N-terminal pleckstrin homology (PH) domain, a phosphotyrosine-binding (PTB) domain and a C-terminal domain (Cossins et al., 2012; Figure 3C). Mutations in *DOK7* underlie a NMJ synaptopathy and comprise a major form of CMS (Beeson et al., 2006).

Structural studies have shown that the PH/PTB domains also mediate DOK7 dimerization, which is necessary to activate MuSK *in vivo* (Bergamin et al., 2010). Interestingly, over-expression of DOK7 in cultured myotubes in the absence of agrin results in full MuSK activation and clustering of AChRs (Inoue et al., 2009), which suggests that DOK7 regulates synapse formation and maintenance by controlling MuSK activity. The C-terminal region of DOK7 has two tyrosine residues, Y396 and Y406, which are phosphorylated by agrin stimulation (Hallock et al., 2010). These residues and their surrounding sequences form Src homology2 (SH2) target motifs that recruit adaptor proteins Crk and Crk-L via their SH2 domains. More recently, adaptor proteins Sorbs1 and -2 have been found to interact with Crk-L and be necessary for AChR cluster formation (Hallock et al., 2016). *In vitro* phosphorylation assays and murine studies have shown that DOK7 C-terminal domain plays a key, but not essential, role in MuSK activation and NMJ development (Ueta et al., 2017). Similarly, patients with DOK7-CMS homozygous for c.1124_1127dupTGCC, which results in a truncated form of DOK7 lacking the SH2 target motifs, have impaired yet active NMJs. However, the selective inactivation of Crk and Crk-L in skeletal muscle causes severe NMJ defects in mice, which suggests that DOK7 could act by two distinct pathways mediated by the N-terminal and C-terminal domains, respectively (Hallock et al., 2010).

Additional players identified in myotubes include Dishevelled-1 (Dvl1) and Tid1, two non-catalytic adapter proteins binding MuSK and contributing in a poorly understood way to AChR clustering. Dvl1 was found to interact with MuSK and regulate AChR clustering through its interaction with downstream kinase PAK1 (Luo et al., 2002) suggesting that agrin may share the signaling pathways of Wnt, which are critical for diverse developmental processes (Komiya and Habas, 2008). However, Dvl1-deficient mice did not show apparent NMJ abnormalities (Lijam et al., 1997) although this could be due to functional redundancy among different Dvl genes. Rac and Rho are monomeric G proteins that link extracellular signals to dynamic changes in the organization of the actin cytoskeleton (Hall, 1998). Rac and Rho play a role in the coupling of agrin signaling to AChR clustering, and in addition, co-expression of constitutively active forms of Rac and Rho can induce the formation of mature AChR clusters when agrin is not present (Weston et al., 2003).

### Rapsyn and Other AchR-Related Proteins

*RAPSN* encodes the 43 kDa receptor-associated scaffold protein of the synapse or rapsyn, which is essential for the postsynaptic specialization of the NMJ (Gautam et al., 1995). Rapsyn is enriched at the postsynaptic membrane, acting as a linker between the AChRs and the cytoskeleton via the dystrophin-associated glycoprotein complex (Apel et al., 1995; Moransard et al., 2003). Early cross-linking studies showed that rapsyn is located in close proximity to the AChR-β subunit (Burden et al., 1983). Subsequently, other AChR subunits have been found to interact with rapsyn in heterologous cell systems (Lee et al., 2010). However, the lack of a crystallographic structure means that the detailed composition of the AChRs-rapsyn network is not well understood (Zuber and Unwin, 2013). Rapsyn is composed of an N-terminal myristoylation moiety (N-Myr) necessary for submembrane localization; seven tetratricopeptide (TRP) domains responsible for rapsyn self-association (Ramarao et al., 2001; Lee et al., 2008); a coiled-coil domain that binds to the cytoplasmic loops of AChRs (Lee et al., 2008), and a RING-H2 domain that links rapsyn to the cytoskeleton through its interaction with the dystroglycan complex (Figure 3D; Apel et al., 1995). *RAPSN* mutations identified in humans are found along the length of the gene and the common p.N88K is located within the TRP domains (Cossins et al., 2006).

Studies in the past have shown that the phosphorylation of the AChR-β subunit mediated by agrin helps to cluster AChRs and anchor them at high density in the postsynaptic membrane, by increasing the stoichiometry of rapsyn/AChR complexes (Borges and Ferns, 2001; Borges et al., 2008). In line with this, mice lacking AChR β-subunit tyrosine phosphorylation develop simplified synapses, although NMJ formation is not compromised (Friese et al., 2007). A recent investigation proposed that the RING-H2 domain of rapsyn contains E3 ligase activity (Li et al., 2016). Another AChR-binding protein recently identified is Vezatin, which is not essential for NMJ formation but may play a role in the formation of postjunctional folds (Koppel et al., 2019).

A dense network of microtubules (MTs) and actin filaments (Dai et al., 2000; Schmidt et al., 2012) interact with the subsynaptic muscle membrane. However, the downstream signals that capture and stabilize microtubules at synaptic AChR clusters are poorly understood. Microtubule actin cross-linking factor 1 (MACF1) has been recently shown to concentrate at the NMJ, where it binds to rapsyn and could serve as an organizer for both actin and microtubule networks (Oury et al., 2019). The study showed that expression at the postsynaptic membrane of microtubule-associated proteins MAP1b, β-TUB, EB1, and Vinculin is MACF1-dependent. MACF1 is not essential for the NMJ formation but postnatal maturation is impaired in *Macf1* mutant mice. Furthermore, two patients from different kinships with CMS have been identified to carry missense variants in *MACF1* (Oury et al., 2019). A second pathway for agrin-induced recruitment of MTs to the postsynaptic membrane is via binding of MTs to CLASP2/CLIP170 (Schmidt et al., 2012).

### ACh-CDK5-Calpain Dispersal Pathway

Muscle depolarization induced by ACh is a negative signal that decreases extrasynaptic AChR concentration by altering the location and stability of AChRs and also by inhibiting their transcription along the muscle fiber (An et al., 2010). This is supported by the findings of increased endplate bandwidth and excessive nerve branching in mice lacking choline acetyltransferase (ChAT), an enzyme required for ACh synthesis (Misgeld et al., 2002). Thus, ACh disperses aneural clusters of AChRs that are not stabilized by agrin signaling. A cyclin-dependent kinase 5 (Cdk5) mechanism (Lin et al., 2005) is thought to drive this pathway through to the interaction of rapsyn and the calcium-dependent protease calpain (Chen et al., 2007; Figure 2). Cdk5 null mice display an abnormally wide central band of AChRs and agrin-induced AChR clustering is markedly increased when Cdk5 activity is suppressed (Fu et al., 2005). The binding of ACh to the AChRs results in increased calcium influx into the postsynaptic membrane that activates calpain activity. Calpain stimulates the cleavage of p35 to p25, a potent co-activator of Cdk5 (Patrick et al., 1999). The ACh-mediated increase in calpain/Cdk5 activity permits cytoskeletal remodeling resulting in the dispersal of AChR clusters (Xie et al., 2006). One study has implicated the intermediate filament protein Nestin in the regulation of Cdk5 activity (Yang et al., 2011). By contrast, agrin is thought to stabilize AChR clusters by promoting the recruitment of calpain to rapsyn and inhibiting calpain activity (Chen et al., 2007). In keeping with the key role of calcium in the dispersion of AChRs, another study showed that blocking dihydropyridine receptors (voltage-gated L-type Ca^2+^ channels) at the muscle membrane resulted in increased MuSK expression, leading to a broad distribution of AChRs and aberrant development of the neuromuscular synapse (Chen et al., 2011). Finally, the suppression of AChR transcription in extrasynaptic regions driven by ACh is thought to be mediated by protein kinase C (PKC) and Ca2+/calmodulin-dependent kinase II (CaMKII) signals resulting in myogenin downregulation (Li et al., 1992; Macpherson et al., 2002). As a helix-loop myogenic transcription factor, myogenin regulates expression of AChRs and other muscle genes by binding the E-boxes located in their promoter and enhancer regions (Eftimie et al., 1991).

### The N-Linked Glycosylation Pathway

**FIGURE 4:**
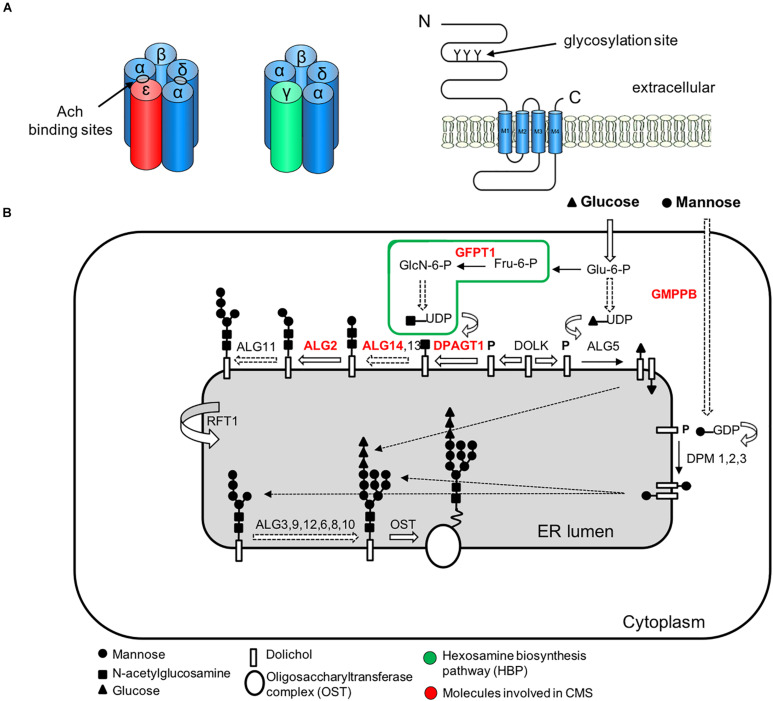
Simplified representation of the AChR pentamer and the *N*-glycosylation pathway of proteins and CMS-related genes (red color). **(A)** Adult (red) and fetal (green) acetylcholine receptors and glycosylation sites. The AChR is made up of five subunits arranged around a central pore. Each subunit is composed of an extracellular domain, four transmembrane domains (M1–M4) and a large cytoplasmic loop linking M3 and M4. Each subunit also contains a conserved N-glycosylation consensus sequence (Asn-X-Ser/Thr) located at the N-terminal region (Gehle et al., 1997), which is necessary for the correct assembly of AChR pentamers and efficient export to the cell surface. **(B)** The *N*-linked glycosylation pathway takes place in the endoplasmic reticulum (ER). The first step is the assembly of the core glycan (*N*-acetylglucosamine, glucose and mannose) on the lipid dolichol. Next, a number of cytosolic glycosyltransferases proceed to dolichol glycosylation on the cytoplasmic face of the ER: GFPT1 synthesizes UDP-GlcNAc (Uridine diphosphate *N*-acetylglucosamine); DPAGT1 and the ALG13/14 complex add the first and second *N*-acetylglucosamine to dolichol. Additional sugar residues are incorporated by ALG2 and other enzymes. Then RFT1 flips the resulting product is flipped into the ER lumen where further sugar moieties are included until the glycan is transferred to asparagine residues of nascent proteins by the multimeric oligosaccharyl transferase complex (OST). DOLK, dolichol kinase; DPM, dolichol-phosphate mannose synthase; Fru-6-P, fructose-6-phosphate; GlcN-6-P, glucosamine-6-phosphate; Glu-6-P, glucose-6-phosphate; GMPPB, GDP-mannose pyrophosphrylase B.

The *N*-linked glycosylation pathway of proteins is a ubiquitous process in eukaryote cells characterized by the sequential attachment of sugar moieties to the lipid dolichol, which is then transferred onto an asparagine residue in a nascent protein (Figure 4). Next-generation sequencing has aided the discovery of an unexpected relationship between myasthenic disorders and defects in the early stages of the *N*-glycosylation pathway (Senderek et al., 2011; Belaya et al., 2012; Belaya, 2015). This highlights that genes with no defined role in neuromuscular transmission can also impair the NMJ structure and function. However, the reasons why defects in a ubiquitous process may result in dysfunction largely restricted to the NMJ are not clear. Glycosylation of AChR subunits is required for the correct assembly of AChR pentamers and efficient export to the cell surface (Gehle et al., 1997). Therefore, abnormal glycosylation results in reduced numbers of AChRs at the muscle endplates, which is most likely the primary mechanism causing impaired neuromuscular transmission (Zoltowska et al., 2013). Other key proteins in NMJ formation and maintenance are also glycosylated, including agrin, dystroglycan, LRP4, MuSK, NCAM and perlecan (Martin et al., 1999). Ultrastructural studies in patients with CMS due to glycosylation defects have shown small sized endplates with simplified postsynaptic regions and poorly developed junctional folds (Belaya et al., 2012; Selcen et al., 2013). In conclusion, while the role of these proteins in the NMJ formation and maintenance is well established, the specific function of adequate glycosylation of NMJ constituents awaits further investigation.

## Extracellular Organizers

The synaptic basal lamina is a specialized form of extracellular matrix containing numerous proteins that are essential for the alignment, organization and maintenance of presynaptic and postsynaptic structures (Sanes, 2003). One of the main components are heterotrimeric proteins of high molecular weight called laminins, which are formed by the incorporation of α, β and γ chains into a cruciform structure and typically self-assemble (Mouw et al., 2014). Of relevance, it was found that soluble laminin can stimulate AChR clustering via dystroglycan independently of MuSK in cultured myotubes (Sugiyama et al., 1997; Montanaro et al., 1998). Furthermore, myotubes cultured on laminin-coated plates (in the absence of agrin) form complex branched AChR clusters similar to those seen *in vivo* following postnatal maturation. Conversely, this process is dependent on MuSK activation (Kummer et al., 2004). Synaptic laminins are also important for presynaptic differentiation as shown in mice lacking laminin β2 (Noakes et al., 1995). Furthermore, laminin α4 has been implicated in the maintenance of the NMJ (Samuel et al., 2012). Overall these studies support the role of laminin as extracellular organizers of the NMJ. Collagen IV is the most abundant protein at the basal lamina. It self-assembles into dimers and hexamers thanks to its globular domains (Mouw et al., 2014). Laminin and collagen networks are connected by Nidogen-2 (Fox et al., 2008; Mokkapati et al., 2008), a non-collagenous glycoprotein, but also anchored to the cytoskeleton by binding to laminin receptors integrin and dystroglycan (Martin et al., 1996). The dystroglycan complex is necessary for the organization and stabilization of the NMJ as shown by the presence of disrupted NMJs in chimeric mice lacking dystroglycan (Côté et al., 1999). Myotubes deficient in dystroglycan are responsive to agrin, but AChR clusters are significantly less stable (Jacobson et al., 2001). By contrast, laminin-induced AChR clusters fail to form in the absence of dystroglycan. Muscle agrin binds to the basal lamina via laminin (Denzer et al., 1997) and α-dystroglycan (Sugiyama et al., 1994), and this is important for the maintenance of the NMJ (Samuel et al., 2012). It is important to highlight that this differs from the role of neuronal agrin in AChR clustering (Ferns et al., 1996). Perlecan, another synaptic heparan sulfate proteoglycan, is linked to both α-dystroglycan and ColQ (Peng et al., 1998). Perlecan-null mice lack AChE at the NMJ confirming its role as an acceptor for collagen-tailed AChE (Arikawa-hirasawa et al., 2002). In addition, muscle-derived COL13A1, which is thought to regulate the maturation of the NMJ (Latvanlehto et al., 2010), has been identified as a CMS-causative gene (Rodríguez Cruz et al., 2019).

## Assembly and Formation of the NMJ

Given that the NMJ is clearly a target for a number of different diseases, the majority of which involve changes in the number or function of specific molecules, it is important to understand fully the way in which this remarkable synapse develops and is maintained. However, it is important to make clear that nearly all current experimental data comes from the study of mice and that observations cannot always be translated to the human NMJ or indeed to other species and mammals (Table 2).

**TABLE 2:** Molecules identified in NMJ formation, maturation and maintenance and levels of evidence.

	Molecule	Cellular level	Animal level	Human disease
NMJ formation	* Agrin	Ferns et al., 1993	Bogdanik et al., 2011	Huzé et al., 2009; Gasperi et al., 2014
	ß-catenin (muscle)	Zhang et al., 2007	Li et al., 2008	N/A
	Calpain	Chen et al., 2007	N/A	N/A
	CaMKII/PKC	Macpherson et al., 2002	N/A	N/A
	Cdk5	Fu et al., 2005	Fu et al., 2005	N/A
	Crk/CrkL	Hallock et al., 2010	Hallock et al., 2010	N/A
	Dihydropyridine-R	N/A	Chen et al., 2011	N/A
	* DOK7	Okada et al., 2006; Cossins et al., 2012	Okada et al., 2006; Arimura et al., 2014	Beeson et al., 2006
	Dvl1	Luo et al., 2002	Lijam et al., 1997	N/A
	* α-dystroglycan	Jacobson et al., 2001	Côté et al., 1999	Belaya, 2015
	Erm	N/A	Hippenmeyer et al., 2007	N/A
	FGF 7/10/22	Fox et al., 2007	Ohno et al., 1999	N/A
	GABP	Schaeffer et al., 1998	Schaeffer et al., 1998	N/A
	* Laminin ß2	Nishimune et al., 2004	Noakes et al., 1995	Matejas et al., 2010
	* LRP4	Zhang et al., 2008	Weatherbee et al., 2006; Yumoto et al., 2012	Higuchi et al., 2011; Ohkawara et al., 2013
	* MuSK	Koneczny et al., 2013	Chevessier et al., 2008; Cole et al., 2008	Hoch et al., 2001; Chevessier et al., 2004
	Myogenin	Eftimie et al., 1991	Arnold and Braun, 1996	N/A
	* N-glycosylation	Belaya et al., 2012; Zoltowska et al., 2013	Issop et al., 2018	Senderek et al., 2011; Belaya et al., 2012; Cossins et al., 2013; Belaya, 2015
	Nestin	Yang et al., 2011	Yang et al., 2011	N/A
	PAK1	Luo et al., 2002	N/A	N/A
	Perlecan	Peng et al., 1998	Arikawa-hirasawa et al., 2002	N/A
	Pro-BDNF	Yang et al., 2009	N/A	N/A
	Rac and Rho	Weston et al., 2003	N/A	N/A
	* Rapsyn	Cossins et al., 2006	Xing et al., 2019	Ohno et al., 2002
	SIRP-α	Umemori and Sanes, 2008	N/A	N/A
	Sorbs1 and Sorbs2	Hallock et al., 2016	N/A	N/A
	Tid1	Linnoila et al., 2008	N/A	N/A
NMJ maturation	* COL13A1	Latvanlehto et al., 2010	Latvanlehto et al., 2010	Logan et al., 2015
	CLASP2/CLIP170	Schmidt et al., 2012	Schmidt et al., 2012	N/A
	Ephexin1	Shi et al., 2010	Shi et al., 2010	N/A
	FGFBP1	N/A	Taetzsch et al., 2017	N/A
	GDNF	N/A	Nguyen et al., 1998	N/A
	Neurofascin 155	N/A	Roche et al., 2014	N/A
	Laminin α4	N/A	Nishimune et al., 2008	N/A
	* Laminin α5	N/A	Nishimune et al., 2008	Maselli et al., 2017
	* MACF1	Oury et al., 2019	Oury et al., 2019	Oury et al., 2019
	MHC1-I	N/A	Tetruashvily et al., 2016	N/A
	NRG1-III	N/A	Lee et al., 2016	N/A
	Vezatin	Koppel et al., 2019	Koppel et al., 2019	N/A
NMJ maintenance	* Agrin	Huzé et al., 2009	Samuel et al., 2012; Tezuka et al., 2014	Huzé et al., 2009
	α-dystrobrevin	Grady et al., 2000	Grady et al., 2000	N/A
	α-syntrophin	N/A	Adams et al., 2000	N/A
	CaMKII	N/A	Martinez-Pena et al., 2010	N/A
	* DOK7	Cossins et al., 2012	Eguchi et al., 2016	Beeson et al., 2006
	Laminin α4	N/A	Samuel et al., 2012	N/A
	* LRP4	Shen et al., 2013	Barik et al., 2014	Ohkawara et al., 2013
	* MuSK	Koneczny et al., 2013	Kong et al., 2004	Hoch et al., 2001
	* N-glycosylation	Belaya et al., 2012; Zoltowska et al., 2013	Issop et al., 2018	Senderek et al., 2011; Belaya et al., 2012; Cossins et al., 2013; Belaya, 2015
	NCAM	N/A	Rafuse et al., 2000	N/A
	Neuregulin/ErbB	N/A	Lin et al., 2000; Fu et al., 2005	N/A
	Neurotrypsin	N/A	Bolliger et al., 2010	N/A
	PKA/PKC	N/A	Martinez-Pena et al., 2013	N/A
	Sarcoglycan-alpha	N/A	Zhao et al., 2018	N/A
	Shp2	Madhavan et al., 2005	Dong et al., 2006	N/A
	Src-family kinases	Smith et al., 2001	N/A	N/A

*Molecules with the strongest evidence are marked with an asterisk; N/A, not available.

**FIGURE 5:**
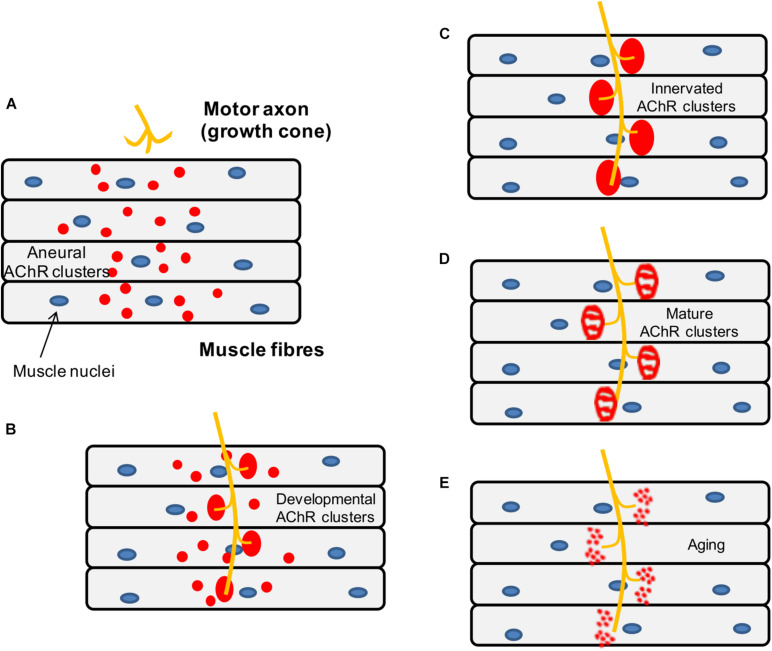
Schematic representation of the mouse NMJ development. **(A)** Aneural AChR clusters are pre-patterned in the midbelly of the muscle fibers prior to the arrival of the nerve terminal. **(B)** Upon innervation of the pre-patterned clusters in the synaptic region, they become enlarged while aneural clusters located in the extrasynaptic region disappear. **(C)** As a result, AChR clusters are concentrated at a high density in the area underneath the nerve terminal maximizing the efficiency of neuromuscular transmission. **(D)** Postnatal maturation of AChR clusters and plaque to pretzel transformation. **(E)** Fragmentation of AChR clusters with aging.

During development, the axons of motor neurons are guided to innervate skeletal muscles (Wu et al., 2010) but it is unclear whether motor neurons or muscles fibers determine the exact location of the endplate band (Figure 5). *In vivo* studies in mice have shown that small AChR clusters are prepatterned in the middle region of the muscle prior to the arrival of the motor axon via a process that requires MuSK and rapsyn but is not dependent on agrin (Lin et al., 2001). Furthermore, it has been shown that this phenomenon also occurs in mutant animals lacking motor nerves, which suggests that pre-patterning is nerve-independent and driven by a muscle-intrinsic program dependent on MuSK (Yang X. et al., 2001).

Subsequently, when the motor neurons innervate some of the prepatterned AChR clusters, these become enlarged and stable while aneural AChR clusters tend to disappear, so that the NMJs are eventually formed in the central region of muscle fibers. As mentioned above, neuronal agrin (McMahan, 1990) drives the process by activating the LRP4-MuSK-Dok7 pathway, which is crucial for the clustering of AChRs underneath the nerve terminal. By contrast, aneural clusters not stabilized by agrin signaling are dispersed by a negative signal, believed to be driven by the release of ACh from the presynaptic terminal (Lin et al., 2005).

Genes coding for components of the neuromuscular synapse become increasingly expressed in subsynaptic nuclei, resulting in the concentration of proteins required locally at the NMJ (Schaeffer et al., 2001), whereas expression of these specific genes is repressed at nuclei elsewhere along the muscle fiber. Even before innervation, AChR gene expression is enriched in the central region of embryonic skeletal muscles (Lin et al., 2001; Yang X. et al., 2001), which suggests that neuronal agrin is dispensable for early transcriptional compartmentalization. However, the area of AChR gene expression in muscles lacking motor axons is wider than usual, pointing that neural signals refine muscle-autonomous prepatterning (Yang X. et al., 2001). One of these signals is likely to be agrin, since compartmentalized expression in subsynaptic nuclei is severely affected in agrin and MuSK deficient mice as shown by *in situ* hybridization experiments to explore the distribution of AChR subunit mRNAs (DeChiara et al., 1996; Gautam et al., 1996). This does not occur in rapsyn deficient mice where AChR genes are selectively expressed by synaptic nuclei in the absence of AChR clusters (Gautam et al., 1995).

The communication between synaptic signals and targeted transcription is thought to be mediated by specific promoter elements in synaptic genes and E-twenty six (ETS) transcription factors. In particular, the N-box, a six-base pair element, was identified as a critical element in targeting the transcription of AChR delta (Koike et al., 1995) and epsilon subunits (Duclert et al., 1996). Disruption of this element in mouse models results in widespread expression of the reporter gene throughout the entire muscle fiber. This was further confirmed following the report of CMS patients underlying mutations in a conserved ETS binding site (N-box) in the promoter region of *CHRNE* encoding the AChR epsilon subunit (Ohno et al., 1999; Abicht et al., 2002). N-box motifs have also been reported to drive synapse-specific expression of utrophin (Gramolini et al., 1999) and AChE (Chan et al., 1999). ETS transcription factors identified in subsynaptic gene expression include GABP (GA-binding protein) α-subunit (Schaeffer et al., 1998) and Erm (Hippenmeyer et al., 2007).

Presynaptic differentiation begins after axon formation and culminates with the assembly of the neuromuscular synapse and the differentiation of a functional nerve terminal opposite the specialized postsynaptic membrane, where presynaptic proteins become concentrated. A relatively recent finding is that LRP4 works as a muscle-derived retrograde signal that controls the early steps of presynaptic differentiation by binding to motor axons and inducing clustering of synaptic vesicles and active zone proteins (Yumoto et al., 2012). Interestingly, although MuSK overexpression in *Lrp4* mutant mice restored AChR clustering, it failed to rescue presynaptic differentiation as motor axons kept growing along the muscle and rarely contacted AChR clusters (Yumoto et al., 2012). This finding suggests that, in addition to its function as a co-receptor for agrin, LRP4 has additional roles in NMJ formation that are independent of MuSK.

Other molecules identified as synaptic organizers of the presynaptic terminal include β-catenin (Li et al., 2008), laminin β2 (Nishimune et al., 2004), fibroblast growth factors (FGF) of the 7/10/22 subfamily (Fox et al., 2007), collagen IV (Fox et al., 2007) and signal regulatory protein α (SIRP-α) (Umemori and Sanes, 2008). However, in the absence of these molecules, motor axons manage to contact AChR clusters and differentiate considerably, which suggests that they act at a later stage in presynaptic differentiation compare to LRP4. Muscle-derived β-catenin, a signaling protein involved in the canonical Wnt pathway, has also been found to regulate motoneuron differentiation as mice lacking muscle β-catenin exhibit AChR clusters distributed throughout a wider region, mislocation of nerve branches and neurophysiological abnormalities (Li et al., 2008). By contrast, there are no obvious NMJ defects in motoneuron-specific β-catenin–deficient mice. Laminin β2, a component of the basal lamina, organizes active zones in motor nerve terminals by binding directly to voltage-gated calcium channels (VGCC) that are required for AChR release (Nishimune et al., 2004). It is thought that this association leads to clustering of VGCC into arrays, which in turn recruit and stabilize other constituents of the presynaptic apparatus. In agreement with this, mice lacking laminin β2 display structural abnormalities at the NMJ, namely a decreased number of active zones (Noakes et al., 1995). Furthermore, mutations in *LAMB2* encoding laminin β2 cause a severe form of synaptic CMS in humans (Maselli et al., 2009).

## Maturation of the NMJ

Neuromuscular junctions are functionally active in the embryonic stage, but they undergo complex postnatal maturation during the first weeks of life including synaptic elimination, endplate remodeling, and the AChR gamma-to-epsilon switch. During this period, the NMJ increases in size and the sarcolemma develops invaginations called postjunctional folds that increase total surface area. The morphology of the NMJ is changed from oval to a more complex perforated plaque, which in mice is described as pretzel-shaped, with a nearly contiguous arrangement of AChRs.

During synapse elimination, all but one axon are gradually withdrawn from multiply innervated muscle fibers, leaving a single innervating axon at each NMJ (Lichtman and Colman, 2000). This is a competitive and asynchronous process taking place at each endplate where more active synaptic sites destabilize neighboring inactive synapses (Balice-Gordon and Lichtman, 1994; Keller-Peck et al., 2001). Although synaptic activity and in particular spike timing seem to drive synaptic elimination (Favero et al., 2012), it is now believed that all three components of the NMJ contribute to this process (Lee, 2020). In particular, tSCs have been found to participate in the pruning of developing synapses through the phagocytosis of immature axons and the displacement of nerve terminals from each other and the postsynaptic membrane (Smith et al., 2013).

A study has identified axon-tethered Neuregulin1 (NRG1-III) as a molecular determinant for tSC-driven synaptic plasticity (Lee et al., 2016). NRG1-III expression coincides temporally with synapse pruning and transgenic manipulation of NRG1-III levels in mice altered the motor input loss rate at NMJs during synapse elimination. However, it is still not clear how this relates to motor neuron activity. Another study showed that loss of glial Neurofascin155 in mice delays developmental synapse elimination by disrupting neuronal cytoskeletal organization and trafficking pathways in motor axons (Roche et al., 2014). On the muscle side, overexpression of glial cell line–derived neurotrophic factor (GDNF) causes hyperinnervation of NMJs in neonatal mice (Nguyen et al., 1998). Other candidates thought to participate in the refining of the neuromuscular circuitry include the major histocompatibility complex, class I (MHC-I) (Tetruashvily et al., 2016), pro-brain-derived neurotrophic factor (pro-BDNF) (Yang et al., 2009) and fibroblast growth factor binding protein 1 (FGFBP1) (Williams et al., 2009).

The remodeling of the murine endplates during early postnatal life results in a plaque-to-pretzel transition where the NMJs become perforated and increasingly complex with multiple branches innervated by a single axon (Slater, 1982). Using cultured aneural myotubes on laminin-coated plates that mimic the *in vivo* transformation, it was shown that perforations in the AChR aggregates bear structures resembling podosomes whose location and dynamics are spatiotemporally correlated with changes in the topology of AChR clusters (Proszynski et al., 2009). Podosomes are adhesive dynamic actin-rich matrix remodeling organelles described in numerous cell types. However, evidence for the relevance of podosomes *in vivo* is scarce and specifically, there is no definitive proof of the existence of synaptic podosomes at the NMJ in living organisms (Bernadzki et al., 2014). Other actors thought to be involved in the plaque-to-pretzel transition are synaptic laminins α4 and α5 (Nishimune et al., 2008) and ephexin1 (Shi et al., 2010). The latter is a rho guanine nucleotide exchange factor (GEF) involved in actin cytoskeletal dynamics. Adult ephexin1^–/–^ mice present with severe muscle weakness, impaired neuromuscular transmission and abnormal maturation of the NMJ into the pretzel-like shape (Shi et al., 2010). Finally, being significantly smaller and more fragmented than murine NMJs (Jones et al., 2017), human neuromuscular synapses may undergo a different process of postnatal maturation.

The adult nicotinic AChR is a pentameric complex composed of four different transmembrane subunits (α-, ß-, δ-, and ε/γ-subunits) (Karlin, 2002; Figure 4). During early postnatal life, the fetal form of the AChR, containing a gamma subunit (2α:ß:δ:γ) is gradually replaced by an epsilon subunit-containing adult form (2α:ß:δ:ε), leading to increased calcium conductance of the receptor (Missias et al., 1996). The half-life of synaptic AChRs also increases during maturation as insertion of new AChRs and the recycling of internalized AChRs maintain the high density of AChRs at the crests of postsynaptic junctional folds (Bruneau et al., 2005; Bruneau and Akaaboune, 2006).

## Maintenance of the NMJ

The apparent macroscopic stability of the NMJ conceals a remarkable molecular dynamism where AChRs are continually exchanged between synaptic and extrasynaptic regions to maintain the high density of AChRs at the postsynaptic membrane (Akaaboune et al., 2002). The homeostasis of the neuromuscular synapse throughout life is essential for the NMJ function, as inactivation of the underlying molecular mechanisms results in synaptic disassembly (Tezuka et al., 2014).

It has been shown using postnatal knockdown experiments that most molecules involved in synaptic formation such as Agrin, MuSK and DOK7 are later required for NMJ maintenance (Kong et al., 2004; Barik et al., 2014; Tezuka et al., 2014; Eguchi et al., 2016). They may also have distinct roles in synapse formation and maintenance: for instance, the forced expression of DOK7 in agrin deficient mice restores synapse formation but NMJs disappear rapidly after birth, which points to an additional role of agrin distinct from MuSK activation in postnatal maintenance (Tezuka et al., 2014). By contrast, other molecules playing an important role in NMJ stabilization and maintenance are dispensable during synapse formation: some components of the dystrophin-glycoprotein complex (DGC) (Ibraghimov-Beskrovnaya et al., 1992), src-family kinases (Smith et al., 2001), NCAM (Rafuse et al., 2000), neuregulin (Schmidt et al., 2011), and more recently, MACF1 (Oury et al., 2019). There is also increasing evidence from clinical (Lashley et al., 2010; Rodríguez Cruz et al., 2015) and experimental studies (McMacken et al., 2019; Vanhaesebrouck et al., 2019) that β2-adrenergic signaling could play a role in NMJ homeostasis. Furthermore, one study in mice proposed that sympathetic neurons make close contact with NMJs (Khan et al., 2016).

The DGC complex links the cytoskeleton of muscle fibers to the extracellular matrix (Ibraghimov-Beskrovnaya et al., 1992). Mice lacking α-dystrobrevin, a cytoplasmic component of DGC, show no abnormalities in NMJ morphology at postnatal day 7. However, by 1 month of age and independently of muscle changes, AChRs became abnormally distributed with irregular branch borders while the size, number and arrangement of branches remained unaltered (Grady et al., 2000). Another study showed that rates of AChR turnover were significantly increased in mice lacking α-dystrobrevin compared to WT and mdx mice (Akaaboune et al., 2002). *In vitro*, α-dystrobrevin is dispensable for agrin-induced cluster formation but required for maintenance of clusters following agrin withdrawal (Grady et al., 2000). A similar phenotype was reported in α-syntrophin null mice in the absence of myopathy (Adams et al., 2000). The structural abnormalities seen in the mdx mouse model of Duchenne muscular dystrophy (Sicinski et al., 1989) are more profound with severe endplate fragmentation (Torres and Duchen, 1987). However, these are found exclusively at NMJs on regenerated muscle fibers, which indicates that endplate remodeling is related to muscle damage rather than dystrophin deficiency (Lyons and Slater, 1991).

Src family kinases (src, fyn, and yes) have been implicated in signaling pathways downstream of MuSK (Fuhrer and Hall, 1996). Studies in *src*^–^*^/^*^–^*;fin*^–^*^/^*^–^ and *src*^–^*^/^*^–^*;yes*^–^*^/^*^–^ mutant mice showed normal NMJ development and agrin-induced phosphorylation of the AChR-ß subunit but AChR clusters in mutant cell lines were significantly less stable following agrin withdrawal (Smith et al., 2001). The neuronal cell adhesion molecule (NCAM) is thought to participate in the maturation of the presynaptic terminal as NCAM null mice present delayed presynaptic structural maturation and smaller endplates (Rafuse et al., 2000). Two serine/threonine kinases, PKC and PKA, have been implicated in the regulation of AChR dynamics at the adult NMJ of living mice by possibly acting on different receptor subunits and/or substrates involved in the anchoring of AChRs (Martinez-Pena et al., 2013). In addition, Ca^2+^/calmodulin-dependent kinase II (CaMKII) is thought to participate in the recycling of AChRs necessary to maintain postsynaptic AChR density (Ibraghimov-Beskrovnaya et al., 1992).

Finally, it is increasingly more evident that glial cells have an important role in NMJ maintenance. Characterization of NMJs after genetic ablation of tSCs in adult mice shows NMJ fragmentation and neuromuscular transmission defects (Barik et al., 2016). In adult frogs, selective ablation of tSCs results in widespread retraction of existing synapses (Reddy et al., 2003). One possible mechanism is through neuregulin/ErbB signaling as *ErbB2*^–^*^/^*^–^ mice lack tSCs and postjunctional folds and although they retain the ability to form neuromuscular synapses, these fail to be maintained (Riethmacher et al., 1997; Lin et al., 2000). Other study in frogs suggested that tSCs express active agrin and enhance aggregation of AChRs on muscle fibers (Yang J.F. et al., 2001).

## Regeneration of the NMJ

Injury to the nerve or muscle, lack of physical activity and ageing can alter synaptic organization resulting in endplate fragmentation, partial denervation and reduction in active zones and AChR density (Stanley and Drachman, 1981; Lyons and Slater, 1991; Valdez et al., 2010).

It is well known that mouse muscle endplates lose the normal pretzel shape and become fragmented with multiple spot contacts following muscle fiber damage (Lyons and Slater, 1991). One of the best examples is the *mdx* mouse model of Duchenne muscular dystrophy (Sicinski et al., 1989). In 8-week old *mdx* mice, muscle endplates from regenerating fibers appear dramatically fragmented over an enlarged postsynaptic area and nerve terminals display abnormally complex features, with a significant increase in the number of fine terminal arborizations, many bearing bouton-like swellings (Lyons and Slater, 1991). The alterations seen in the *mdx* mouse increase with ageing, probably as a consequence of recurrent muscle damage (Torres and Duchen, 1987). Interestingly, the structural changes do not alter the safety margin of neuromuscular transmission (Nagel et al., 1990). Therefore, the significance of this process is not fully understood as this could be the outcome of a physiological mechanism of NMJ maintenance rather than synapse degeneration (Slater, 2019).

Endplate reinnervation following nerve injury results in degradation of junctional AChRs, increase of their turnover rates and structural changes to the NMJ (Stanley and Drachman, 1981; Rich and Lichtman, 1989; Akaaboune et al., 1999). Upon denervation, it is thought that the upregulation of chromatin acetylation and AChR expression is mediated by signal-responsive transcriptional regulators histone deacetylase 9 (HDAC9) and 4 (HDAC4) that allow the induction of the transcription factor Myogenin (Méjat et al., 2005; Cohen et al., 2007). Protein kinase B (PKB/Akt) and mTORC1 (mammalian Target of Rapamycin Complex 1) have been implicated in regulating muscle homeostasis and maintaining NMJs after nerve injury in mice (Castets et al., 2019). Other key elements of synapse development such as Col13a1 (Zainul et al., 2018) and LRP4 (Gribble et al., 2018) have also been involved in promoting peripheral nerve regeneration. Finally, tSCs play a key role in reinnervation by guiding axons through the extension of their processes (Son and Thompson, 1995) and retracting processes from territory they previously occupied within the endplate (Kang et al., 2014).

## Author Contributions

PR reviewed the literature and drafted the manuscript. JC and DB contributed to critical revision of the manuscript for important intellectual content. AV contributed to critical revision of the manuscript for important intellectual content and senior authorship. All authors contributed to the article and approved the submitted version.

## Conflict of Interest

University of Oxford and AV hold patents and receive royalties for antibody assays from Euroimmun AG and Athena Diagnostics. The remaining authors declare that the literature review was conducted in the absence of any commercial or financial relationships that could be construed as a potential conflict of interest.
